# Porous Alumina Ceramics with Multimodal Pore Size Distributions

**DOI:** 10.3390/ma14123294

**Published:** 2021-06-14

**Authors:** Jonas Biggemann, Martin Stumpf, Tobias Fey

**Affiliations:** 1 Department of Materials Science (Glass and Ceramics), University of Erlangen-Nuernberg, Martensstr. 5, D-91058 Erlangen, Germany; jonas.biggemann@fau.de (J.B.); martin@stumpfteam.de (M.S.); 2 Frontier Research Institute for Materials Science, Nagoya Institute of Technology, Gokiso-cho, Showa-ku, Nagoya 466-8555, Japan

**Keywords:** digital-twin, volume-of-interest, hierarchical network, sacrificial template, multimodal pore size distribution

## Abstract

Pore networks with multimodal pore size distributions combining advantages from isotropic and anisotropic shaped pores of different sizes are highly attractive to optimize the physical properties of porous ceramics. Multimodal porous Al_2_O_3_ ceramics were manufactured using pyrolyzed cellulose fibers (l = 150 µm, d = 8 µm) and two types of isotropic phenolic resin spheres (d = 30 and 300 µm) as sacrificial templates. The sacrificial templates were homogeneously distributed in the Al_2_O_3_ matrix, compacted by uniaxial pressing and extracted by a burnout and sintering process up to 1700 °C in air. The amount of sacrificial templates was varied up to a volume content of 67 Vol% to form pore networks with porosities of 0–60 Vol%. The mechanical and thermal properties were measured by 4-point-bending and laser flash analysis (LFA) resulting in bending strengths of 173 MPa to 14 MPa and heat conductivities of 22.5 Wm^−1^K^−1^ to 4.6 Wm^−1^K^−1^. Based on µCT-measurements, the representative volume-of-interest (VOI) of the samples digital twin was determined for further analysis. The interconnectivity, tortuosity, permeability, the local and global stress distribution as well as strut and cell size distribution were evaluated on the digital twin’s VOI. Based on the experimental and simulation results, the samples pore network can be tailored by changing the fiber to sphere ratio and the overall sacrificial template volume. The presence pore formers significantly influenced the mechanical and thermal properties, resulting in higher strengths for samples containing fibrous templates and lower heat conductivities for samples containing spherical templates.

## 1. Introduction

Advanced porous ceramics show excellent high-temperature stability, corrosion resistance, high strength and hardness combined with the structural advantages of porous architectures such as low thermal conductivity, adjustable permeability, and high surface area [1,2,3]. These remarkable structural features enable a broad range of engineering applications such as ceramic filters, catalysts, light-weight components, thermal insulators, and bioceramics [1,2,3,4,5,6]. Traditionally, pores have been completely avoided in load-bearing components based on their negative impact on the mechanical properties, acting as critical flaws for catastrophic brittle failure [2,6]. However, the introduction of oriented, tailored porosity can be advantageous to fabricate strain tolerant lightweight structures with high specific strength and reduced stiffness [3,7]. Porous ceramics with low elastic moduli are highly attractive for high temperature applications based on their outstanding thermal shock resistance [3] and may also be beneficial for bioceramic implants with reduced stress shielding [8,9].

The structural and functional features of porous ceramics are predominantly influenced by the nature of their pore network, which is characterized by the total amount of porosity, pore size, distribution, and interconnectivity [1,5,6]. It is the ultimate goal in porous ceramic processing to tailor the pore network for specific applications. Among the various manufacturing techniques, sacrificial templating is the most straightforward technique to obtain customized pore structures with a broad porosity range (20–90 Vol%) and variable pore sizes (1–700 µm) [6]. During sacrificial templating, fugitive additives are dispersed in a ceramic matrix and afterwards extracted via burnout, evaporation, or dissolution to generate pores. The extraction of the pore-formers leaves the negative replica of the sacrificial template inside the ceramic structure.

The pore network can deliberately be tuned by the amount, size, shape, and morphology of the sacrificial templates [5,6,10]. A great variety of organic pore formers including micro spheres (PMMA [10,11,12], PVC [13], PS [14], PE [15]), natural templates (starch [10,16], cotton [17]) and fibers/whiskers (Nylon [18], Rayon [19,20], cellulose [21,22], and CNTs [10]) were already successfully incorporated in ceramic matrices. Monodisperse, statistically distributed pores can be generated using isotropic spheres with a geometrical aspect ratio of l/d = 1 [11,12,13,14,15]. Unidirectional oriented pores, as well as continuous pore channels, can be realized by the addition of anisotropic pore formers (l/d >> 1) such as fibers, whiskers, or threads [17,18,19,20]. An autonomous self-alignment of the anisotropic pore formers can be achieved by applying external (shear-) stresses, which commonly occur in conventional shaping technologies such as extrusion [19,20], tape casting [23], and uniaxial pressing [21,22].

A multimodal pore architecture, consisting of both isotropic pore formers for well-defined pore size distributions and anisotropic oriented pore formers connecting isolated spherical pores will modify material properties such as strength, thermal conductivity, and permeability. So far, only a few multimodal sacrificial pore networks were microstructurally investigated [10,15,24,25]. However, the relation between the 3D pore architecture and the resulting material properties and pore networks still has to be characterized. A complete understanding of the 3D oriented pore structure, including interconnectivity, tortuosity, permeability, pore throats and cell windows and local/global stress distribution is mandatory to allow inference on the fluid flow through the pore network.

In this work, multimodal pore networks were fabricated via sacrificial templating using isotropic phenolic resin microspheres and anisotropic pyrolyzed cellulose fibers. The total amount of sacrificial templates was varied between 0 and 67 Vol% with different sphere to fiber ratios to generate interconnecting pore networks and investigate the microstructural influence on the permeability, mechanical, and thermal properties. Emphasis was placed on the digital twin by X-ray micro-computed tomography (µ-CT) and its representative VOI to characterize the 3D pore network combined with microstructural SEM analysis and FEM simulations.

## 2. Materials and Methods

### 2.1. Fabrication of Alumina with Multimodal Pore Size Distribution

**Figure 1:**
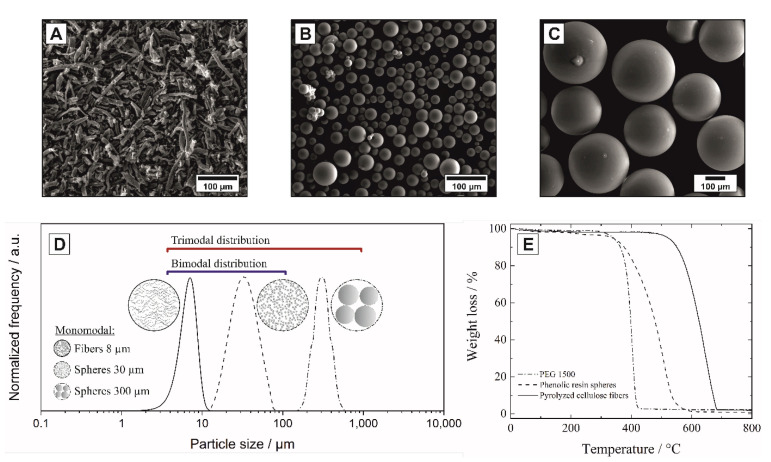
Characteristics of the utilized isotropic and anisotropic sacrificial templates: SEM micrographs (**A**–**C**) of the pyrolyzed cellulose fibers (**A**), the 30 µm (**B**) and 300 µm phenolic resin spheres (**C**); corresponding monomodal particle size distributions with examples for bi-/trimodal mixtures (**D**) and TGA analysis of the template burn-out in air (**E**).

Porous alumina ceramics with multimodal pore size distributions were prepared from an ultrafine α-Al_2_O_3_ powder (CT 3000 SG, Almatis GmbH, Ludwigshafen, Germany, d_50_ = 400 nm) loaded with varying amounts of pyrolyzed cellulose fibers (anisotropic) and phenolic resin spheres (isotropic). The pyrolyzed cellulose fibers were obtained by pyrolyzing cellulose paper (200 g/m^2^, Hahnemühle Fineart GmbH, Dassel, Germany) at 800 °C for 1 h under N_2_-atmosphere. The individual fibers were separated by chopping at 20,000 rpm for 15 s (A10, IKA-Werke GmbH&Co. KG, Staufen im Breisgau, Germany). The diameters and lengths of the pyrolyzed cellulose fibers were optically determined by analyzing SEM micrographs (ESEM, Quanta 200 FEG, FEI Company, Peabody, MA, USA) using ImageJ v1.50i [26]. After chopping, fibers with a mean diameter of d_50_ = 8 µm and length of l_50_ = 150 µm (l/d = 19) were obtained. Phenolic resin spheres (Brace GmbH, Karlstein am Main, Germany) with a mean size of 30 µm and 300 µm determined by laser light scattering (Mastersizer Hydro 2000S, Malvern Instruments GmbH, Herrenberg, Germany, solvent 2-propanol) were used as isotropic templates (l/d = 1). The SEM-micrographs of the isotropic and anisotropic templates, the corresponding template size distributions and TGA curves for the burnout in air are shown in Figure 1. The templates provide hierarchical stages of porosity in the range 1–400 µm with three discrete, monomodal particle sizes.

Al_2_O_3_ powder blends with mono-, bi-, and trimodal mixtures of pyrolyzed cellulose fibers and phenolic resin spheres were homogenized for 30 min by dry mixing (AR 400, ERWEKA GmbH, Heusenstamm, Germany) using polyethylene glycol as the binder (PEG 1500, Merck KGaA, Darmstadt, Germany), as shown in Table 1. The powder mixtures were then uniaxially compacted to plate geometry (50 mm × 50 mm × 5 mm) using a pressure of 53 MPa (PW 10 E, Paul-Otto Weber GmbH, Remshalden, Germany). The subsequent burnout of the sacrificial templates was carried out up to 570 °C for 2 h in air. The heating rates for the burnout process were established using TGA analysis (STA 429, Netzsch Instruments, Selb, Germany) of the binder and templates applying heating rates of 5 K/min, Figure 1E. Subsequently, the samples were sintered at 1700 °C for 2 h.

### 2.2. Mechanical and Thermal Properties Characterization

**Figure 2:**
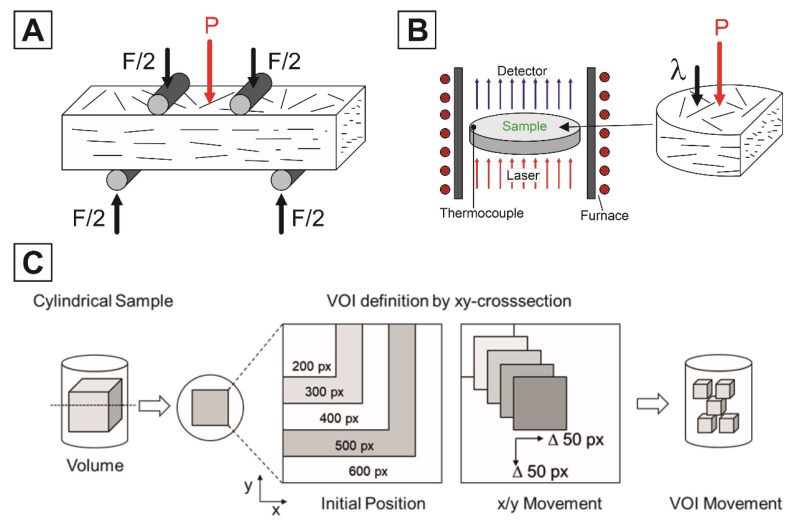
Schematic characterization techniques utilized to determine the physical properties considering the sample orientation and fibrous pore alignment: flexural strength determined by 4-point bending (**A**), thermal conductivity by laser-flash analysis (**B**), and the definition of the analyzed Volume-of-Interests based on the increasing and shifting arguments of the starting volume used for the μCT-analysis (**C**).

Rectangular bars with dimensions of 2.5 mm × 2 mm × 25 mm were prepared by diamond cutting to measure the bending strength by 4-point-bending with a support distance of 20 mm according to DIN EN 843-1 (EXAKT 6000 EA, EXAKT Advanced Technologies GmbH, Norderstedt, Germany), Figure 2A. A constant crosshead speed of 0.5 mm/min was applied. The Young’s modulus was determined by impulse excitation according to DIN EN 843-2 (Buzz-o-sonic 5.9, BuzzMac International LLC, Glendale, CA, USA) using a condenser microphone (Audix TM-1, Audix Microphones, Wilsonville, OR, USA). The thermal conductivities and heat capacities were determined on drilled samples with a diameter of 12.7 mm and thickness of 2 mm by laser flash analysis in flowing argon atmosphere up to 900 °C (LFA 457 Microflash, Netzsch Instruments, Selb, Germany), Figure 2B. For the heat capacity calculation, a pyroceram 9606 standard and the Netzsch Proteus Software (Version 6.0, Netzsch Instruments, Selb, Germany) were used. The samples were coated with a thin graphite layer before the measurements.

### 2.3. Microstructural Characterization and Digital Twin

The microstructure of the multimodal porous alumina was analyzed on fracture patterns or polished cross sections using an environmental scanning electron microscope (ESEM, Quanta 200 FEG, FEI Company, Peabody, MA, USA).

To evaluate the 3D microstructures and pore networks, µCT scans were performed with a Skyscan 1172 (Skyscan, Kontich, Belgium) equipped with a tungsten tube (λ = 0.024 nm) and an 11 MP detector. The µCT scan was performed with a resolution of 2.24 µm/pixel, since the minimum fiber diameter after carbon burnout is about 8 µm and thus can be represented by at least three pixels. The scan resolution and scan time define the maximum sample diameter for the examination, which in this setup is approx 3 mm for rotationally symmetric samples. This ensures that a sampling of 3D volumes is not influenced by edge effects (beam hardening). The specimen height is max. 4 mm. The spacing of the image slices corresponds to the resolution and thus the voxel size is 2.24 × 2.24 × 2.24 µm^3^. The sample was rotated 180° with a rotation step size of 0.2° and an Al filter was used. The 2D sinograms were reconstructed using NRecon (Version 1.6, Skyscan, Kontich, Belgium) and visualized in Amira (Version 2020.2 FEI Imaging Systems, Berlin, Germany). These 3D volume data from the µCT-scans serve as the basis for the creation of the digital twin.

Due to the heterogeneous microstructure of the porous ceramics, the corresponding representative volume-of-interest (VOI) has to be determined for the creation of a digital twin that can be used for further simulations and calculations. Alokar et al. have tried to determine this as a function of the total porosity of the considered volume within a deviation bandwidth (±5%), however this is not transferable to structures with extremely heterogeneous pores (dimension, shape, and length/diameter ratio) and porosities >20% [27]. To enable a systematic selection or determination of the representative volume in heterogeneous porous structures, the consideration of stereological parameters within the microstructure is relevant. For this purpose, approaches to stereological description can be found in DeHoff [28], where the global metric descriptors are explained via stereological relations by Hadwiger [29], DeHoff [28,30], and Ohser and Nagel [31]. Here, the porosity is described via the volume fraction (M0), the mean chord length via the interface density (M1) and the Jeffrey size via the mean curvature integral density (M2) and the curvature integral density by the Euler-Poincaré approach as Euler number (M3). The three descriptors are known as the Minkowski-functionals M1, M2, and M3. The first approaches to determine the stereological parameters in ceramic materials can be found in [32,33], but only for a small number of layers. In the present samples, the determination of the representative VOI and thus of the digital twin was performed using the stereological approaches according to DeHoff and Ohser [28,31]. For this purpose, a 3D volume of 600 × 600 × 600 px was extracted from the samples based on the µCT scans per sample. This corresponds to 1.34 × 1.34 × 1.34 mm^3^, the specification in px is retained for simplicity, since the image evaluation with regard to the stereological parameters is based on the image pixels. This 3D volume was now divided into sections with ascending size starting from 200× 200 × 200 px in 100 px-steps per axis up to the final size. These sections were then shifted in the x-y direction by 50 px each and also in the z-direction by 50 px up to the point that the section still lies within the original volume (see Figure 2C).

On the basis of the sections, the stereological parameters after setting the threshold (material/air) were determined using our own “Practical Extraction and Reporting Language” (PERL) scripts implementing the mathematic description of DeHoff, Ohser, Nagel and Hadwiger [28,29,30,31] and structural parameters such as structure model index (SMI), cell and bar size distribution were calculated using CTAnalyzer, Skyscan, Belgium using the common determination by Odgaard and Hildebrand [34,35,36]. An increase in volume up to the final volume leads to a significant reduction in the error bars and thus to the occurrence of saturation of the stereological parameters. By forming the first or second derivative for the respective parameters, the corresponding curve minima could be determined. These points then represent the minimum representative VOI.

The pore network is determined on the representative VOI by applying skeletonization algorithms with Amira software (ThermoFisher Scientific, Waltham, MA, USA) [37,38]. This can be separated into two steps: (a) calculating the distance map of the thresholded image and (b) using thinning operations on the volume to remain a string of connected pixels. The pore network is divided into cylindrical segments (length, diameter, volume, spatial orientation (theta, phi)) and the nodes (dead node, branch node, end node) are determined with their respective connectivity. Based on histogram distributions of segment diameters/lengths as well as information about the number of connections per node, the microstructure can be described exactly and reliable statements about connectivity can be made. Based on the pore network, the absolute permeability and tortuosity can then be calculated as a function of the input pressure and the entry point, respectively. The input pressure was set to 130,000 Pa, the output pressure to 100,000 Pa, fluid viscosity to 0.001 Pa·s and the pressure direction was parallel to *z*-axis. The representative VOIs were exported from Amira software in STL format and prepared for the FE meshes using the open-source software Meshlab software [39]. This includes the cleanup of defects such as holes, non-manifold edges and vertices, self-intersecting faces, small disconnected components, and empty faces. Partially, this required complex runs with dedicated processing of the corresponding defects. The repaired model was then fully remeshed using the Surface Reconstruction Screened Poisson Algorithmus. A few new defects were unavoidable in the process, which were corrected after the remeshing. The export continued as an STL file. The software Marc.Mentat 2017, (MSC.Software, Munich, Germany) was used for the FE calculations. For the generation of the tetrahedral FE mesh, the import was done as surface mesh and corresponding cleanup of duplicate nodes. Residual nodes were continuously removed by sweeping to achieve an outside edge length = 0. The volume meshing is done with the integrated Patran mesher. The volume in the model was assigned the material properties of Al_2_O_3_ bulk (Young’s modulus 410 GPa, v = 0.22, r = 3.98 g/cm^3^), as boundary conditions a displacement in y = −0.1, which corresponds to a compression (compressive load) of max. 0.224%. The opposite nodes on the model surface were fixed in motion (D_x_ = D_y_ = D_z_ = 0) to represent the loading of a compression test in *y*-axis. The 4-node tetrahedral elements were assigned the computational properties of 3D solid element No. 157 (MSC.Menat VOL B, Elementlibary, MSC.Software, Munich, Germany). The non-linear calculation is performed using the internal direct solver. From the calculation results, the maximum stress occurring at the applied deformation was determined, where the stress distribution occurring is inversely proportional to the strength: high stress values correspond to low strengths.

## 3. Results and Discussion

### 3.1. Microstructural Characterization—SEM and µCT

Macroporous alumina samples with multimodal pore size distributions within a porosity range of 2.3 to 59.1 Vol% were obtained after sintering at 1700 °C, as shown in Table 1. The target porosity of each sample was calculated from the volume fractions of the individual pore-formers, which were varied over a broad range of 0 to 67 Vol% with different modularity and sphere to fiber ratios. With a mean deviation of 1.3% the target porosity is in a good agreement with the experimentally derived total porosity, allowing a simple process control of the resulting total sample porosities.

**Figure 3:**
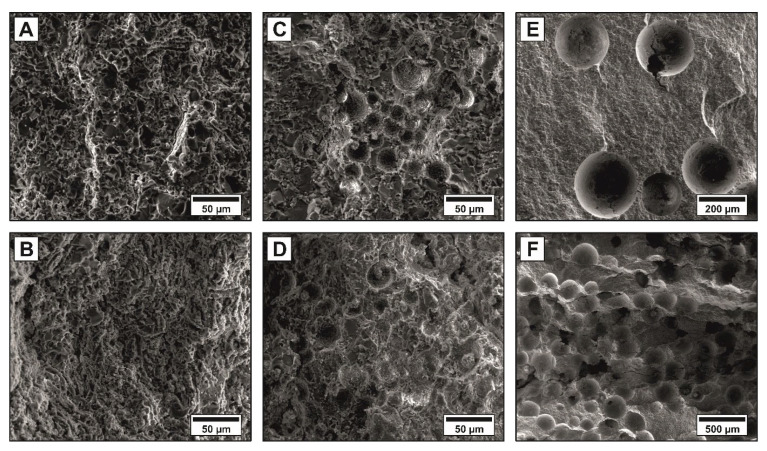
Microstructure of the monomodal samples: SEM micrographs of the typical fracture surfaces of sintered Al_2_O_3_ ceramics containing monomodal distributions of pyrolyzed cellulose fibers (**A**,**B**), 30 µm phenolic resin spheres (**C**,**D**) and 300 µm phenolic resin spheres (**E**,**F**).

The pore morphologies of the alumina ceramics with monomodal distributions of pyrolyzed cellulose fibers are shown in Figure 3A,B, whereas Figure 3C–F show the use of 30 µm (C,D) and 300 µm (E,F) phenolic resin spheres as pore formers. Small amounts of spherical templates lead to the formation of isolated pores as shown in Figure 3E. Agglomerates occasionally occurred at low contents of 30 µm spheres caused by the dry mixing process of the powder and pore formers, as shown Figure 3C. Homogenously distributed spherical pore formers can be ensured by using wet mixing processes [40]. However, in this work a dry mixing process is essential to prevent a swelling of the pyrolyzed cellulose fibers in water or other organic solvents [41,42]. Higher amounts of spherical templates lead to the generation of connected pore networks, as shown in Figure 3D,F, where only a few walls between the pores could be determined (see arrows in the figure). The maximum proportion of pore formers, that still allows for handling and debinding in terms of process technology is shown for the respective pore former type in Figure 3B,D,F.

**Figure 4:**
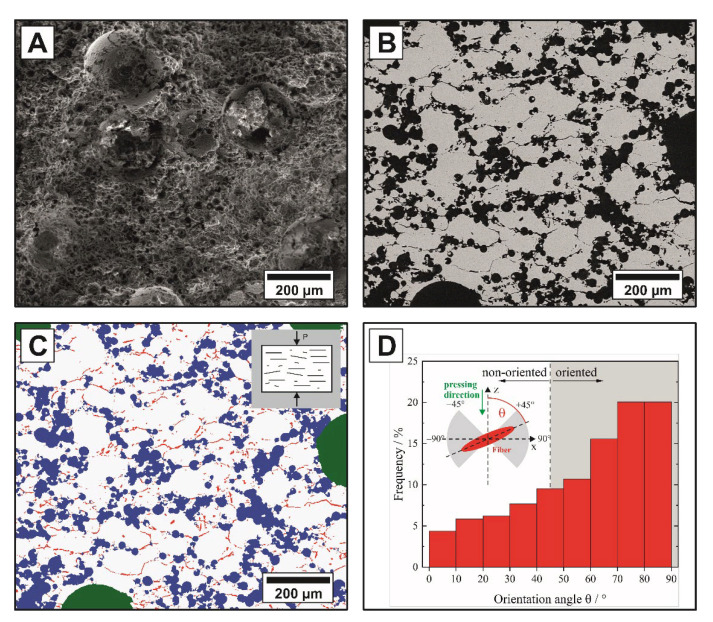
Microstructure of a trimodal sample: SEM micrographs of the fracture surface (**A**) and polished cross-section (**B**) of an alumina ceramic with a trimodal distribution of sacrificial templates (batch No. 13); (**C**) shows the false-color image of the polished cross-section highlighting the interconnected pore network in red (pyrolyzed cellulose fibers), blue (30 µm phenolic resin spheres), and green (300 µm phenolic resin spheres). (**D**) shows the 2D analysis of the fibrous pore orientation represented by the corresponding angular distribution.

Figure 3A shows the pore channels generated by the pyrolyzed cellulose fibers. With an increasing amount of fibers, an interconnected pore network was formed and a pronounced fissuring of the fracture surface occurred, as shown in Figure 3B. A preferential alignment of the fibrous pores could be observed in the polished cross-sectional 2D SEM micrographs for all samples containing pyrolyzed cellulose fibers, including multimodal samples. Due to the three-dimensional arrangement and volume of the pore formers, the determination of orientation, size and shape on polished 2D cross-sectional images represents only a snapshot of this 3D structure, commonly known as the “tomato salad problem”, which can only be solved by a 3D volume observation. Since in general the possibilities of a complete 3D analysis are not always available, in the following both methods are applied to the samples in order to determine the degree of agreement and thus to underline the validity of the 2D method for the samples investigated. An exemplary 2D-evaluation of the microstructure and pore alignment is shown for a trimodal sample (batch No. 13) in Figure 4. The multimodal pore network is characterized by well-distributed 300 µm (green) and 30 µm (blue) spherical pores, which are connected via a fibrous pore network (red), Figure 4A,C. While the isotropic spherical pores (l/d = 1) showed no deformation or orientation after the shaping and sintering process, a pronounced orientation of the tubular pores generated by the anisotropic pyrolyzed cellulose fibers (l/d = 19) was observed. The degree of the fibrous pore alignment was evaluated using a best-fit ellipse approach (Image J 1.50i) as described by Heunisch et al. [43]. Orientation angles (θ) of |±45°| < θ < |±90°| were considered as perpendicular orientated to the pressing direction (see grey section Figure 4D). Figure 4D shows the angular distributions of the fibrous pore network. The fibrous pores show a preferential alignment perpendicular to the pressing direction of 71.5% (+45° < θ < +90°), which can be attributed to an autonomous alignment of the anisotropic pyrolyzed cellulose fibers in the shear gradient during uniaxial pressing [21,22,44,45,46]. The degree of the fiber orientation strongly depends on the fiber content [44] and aspect ratio (l/d-ratio), the densification method (uniaxial dry pressing followed by pressureless sintering, hot pressing, or spark plasma sintering) and the applied pressure [44,45]. Higher pressures might achieve higher degrees of orientation, however, uniaxial pressing limits the orientation of anisotropic templates (fibers, whiskers, platelets) perpendicular to the pressing direction. Parallel to the pressing direction the fibers are randomly oriented [21,22,45,46]. The addition of rigid spherical templates does not interfere with the fiber orientation, as shown in Figure 4B,C. Anisotropic fibers offer a very attractive potential to generate oriented connecting pore channels between larger spherical templates.

**Figure 5:**
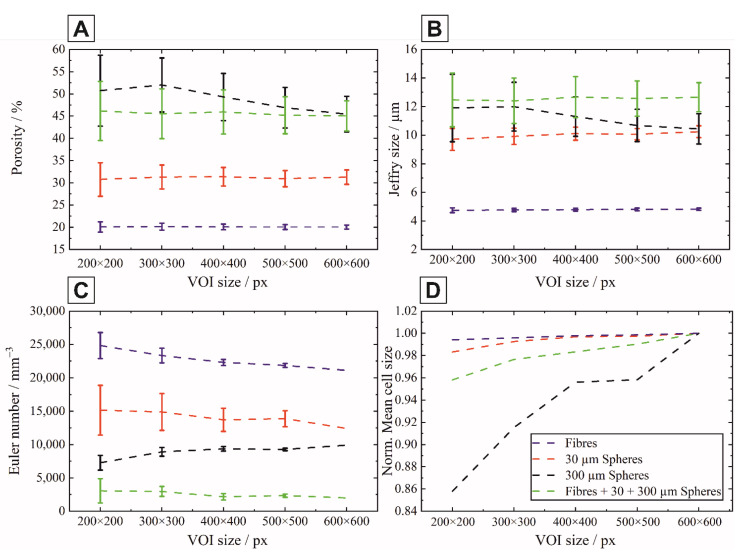
Evaluation of the Minkowski functionals and mean cell size for determination of the representative Volume-of-Interest (VOI). Porosity (**A**), Jeffry size (**B**), Euler number (**C**) and Normalized Mean cell size (**D**) in dependence of the examined VOI between 200 × 200 and 600 × 600 px.

The size quantification of spherical and fibrous pores represents a major challenge as conventional techniques such as mercury intrusion or 2D SEM image analysis tend to underestimate the actual pore sizes since only cell windows (largest pore entrance) and random 2D-sections can be measured [47]. In this work, the alumina ceramics with multimodal pore size distributions were therefore investigated by µ-CT, providing an accurate determination of the spatial extent and orientation in all three spatial directions. Due to the limitation of sample size by the resolution of the µCT equipment and the computational capacity regarding the number of nodes and faces, the representative volume-of-interest was determined by the Minkowski functionals combined with the mean cell size as shown in Figure 5. The number of nodes and faces was determined by the Minkowski functionals. A total of 45 different constellations were examined, 19 for the 200 × 200, 16 for the 300 × 300, 8 for the 400 × 400, 3 for the 500 × 500, and 1 for the 600 × 600 variant. The porosity, plotted in Figure 5A, shows a continuous decrease of the error bars with increasing test volume for all four samples. The porosity error bars vary by ±max 5%, except for the sample with the 300 µm spheres, where this value increases to ±10%. From a VOI of 400 px, a reduction of the error bar takes place, which is consistent with the minimum from the first and inflection point in the second derivative. For the Jeffry size (second Minkowski functional) in Figure 5B, the error bars also decrease, and the Jeffry size for the fiber sample remains unchanged in contrast to the sample with the 30 µm spheres and the trimodal distribution, where an increase of about 5% of the Jeffry size occurs. The different microstructures shown in Figure 3 are also well represented by the Jeffry size. In particular, it can be seen that the samples with spheres (regardless of proportion and size) are close to each other and the fiber sample deviates from them due to the different microstructure and connectivity. The evaluation of the Jeffry size resulted in a minimum and thus representative VOI of 400 px. The connectivity is represented by the third Minkowski functional, the Euler number. Its variation for the different structures is shown in Figure 5C. Corresponding to the porosity and the Jeffry size, the error bars decrease with increasing VOI size. The pure fiber sample exhibits the highest connectivity, which is due to the significantly smaller fiber diameter compared to the spheres. The difference in sphere diameter for the same total volume fraction results in higher connectivity for the 30 µm spheres than for the 300 µm spheres. The trimodal distribution has a significantly lower percentage of fibers as well as spheres and thus the lowest Euler number. The change in Euler number with the increase in VOI (increase or decrease) is due to the structural changes in the heterogeneous samples. However, above a VOI size of 400 px, the variations decrease, and the differences are much smaller. This is also confirmed by the evaluation of the derivations. In addition, the mean cell size was determined. In contrast to the Minkowski functionals, which are derived from stereological and topological parameters, the estimation of the cell size is done via an iterative process, where the diameter of the largest sphere filling the pore is determined. This then results in a pore size distribution that can only be approximated by a Mean cell size in order to compare the structures. In particular, edge pores, which are not completely mapped, can lead to deviating, smaller values here. Since the number of pores and thus the volume is directly included in the calculation, the comparison is made using a normalized mean cell size, where the values for the VOIs smaller than the final volume are normalized by the final volume, as shown in Figure 5D. For diameters <30 µm (spheres and fibers), their course decreases by max. 0.02 with reduced VOI from 600 × 600 to 200 × 200. For the trimodal specimen, the decrease is 0.04, with a linear trend starting at 300 px. The 300 µm spheres sample shows that the mean cell can only be seen as a supplement to the Minkowski functionals. Due to the high scatter of the individual distributions (not shown here), consolidation only occurs from a VOI of 400 × 400 px and above. Assuming a target corridor of ±5% deviation (= 95% interval) from the normalized value, the value for a VOI of 400 px lies within the target range. Due to their heterogeneity and a factor of 10 difference in the diameter of the pore formers, the significance of the mean cell size is lower compared to the Minkowski functionals.

By means of the three Minkowski functionals, which allows for a reduction of the complex microstructure to singular numerical values, the representative VOI could be determined to 400 px for the present samples.

**Figure 6:**
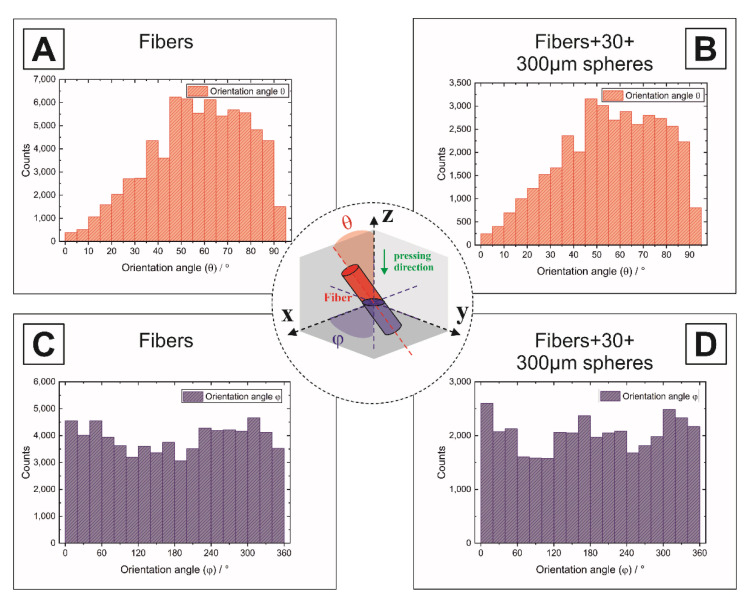
Orientation of the fibrous pores in the 3D volume analyzed by µCT: (**A**,**B**) show the fibrous pore orientation (angle θ) towards the *z*-axis, (**C**,**D**) show the fiber orientation (angle φ) within the xy-plane.

Based on the representative VOI, the digital twin can be used to determine the 3D fiber orientation in the volume. Here, the polar angle theta (θ) between the *z*-axis and xy-plane is determined in the range 0–90° measured from the *z*-axis towards the xy-plane as well as the angle phi (φ) in the range 0–360° within the xy-plane, see Figure 6. A theta angle of 0° corresponds to a parallel direction to the *z*-axis and of 90° parallel to the xy-plane. The angular distributions of Figure 6A,B prove that more than 60% of the fibers are oriented perpendicular to the pressing direction, shown by angles greater than 45°. The results from Figure 6A,B underline the result of the 2D analysis from Figure 3 and supplement this with a complete observation from 3D volumes. The orientation in the xy-plane shows for the pure fibers in Figure 6C that there is no preferred direction, i.e., the fibers are oriented randomly and thus a heterogeneous microstructure is present. The multimodal samples (Figure 6B,D) show no significant difference in the orientation, proving the observations of the SEM that the fiber orientation is not effected by the addition of spherical pore formers.

**Figure 7:**
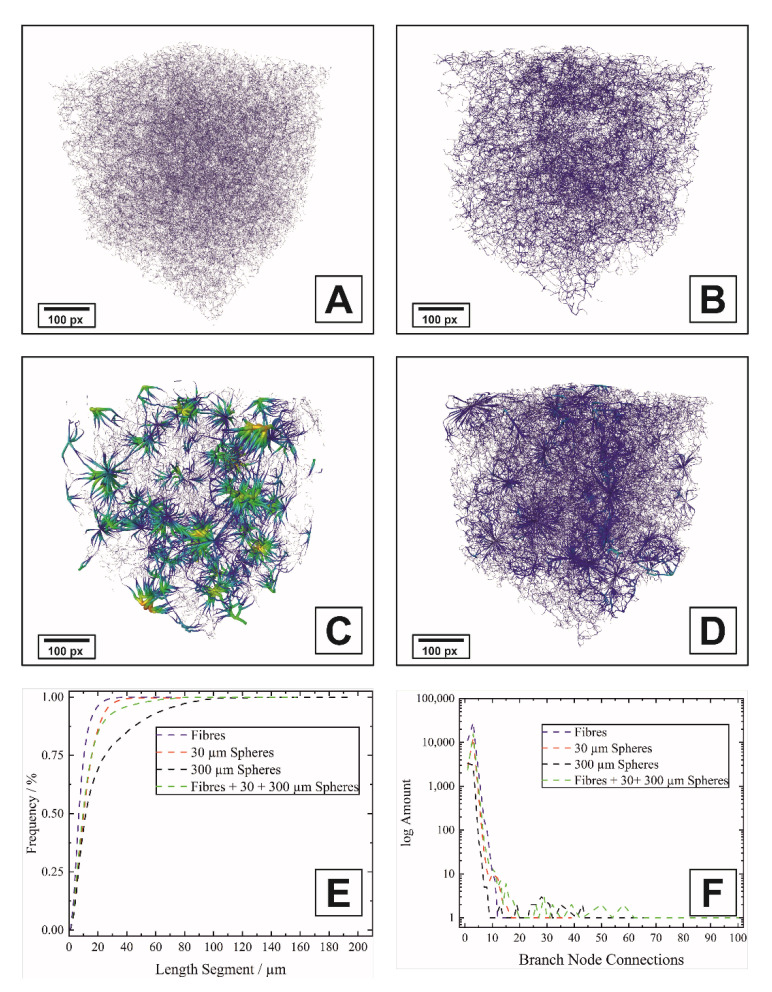
Pore network of monomodal samples with fibrous pores (**A**), 30 µm (**B**) and 300 µm spherical pores (**C**) and trimodal sample containing all type of pore formers (Batch No. 13) (**D**). For figure (**A**–**D**), the color indicates the pore diameter, minimum blue ≤5 µm, maximum red ≥60 µm. (**E**) shows the sum curves of the length distribution of the segments and (**F**) the amount of connectivity per branch node.

Furthermore, the digital twin was used to determine the pore network of the different microstructures, see Figure 7. If only fibers are used as pore formers (Figure 7A), the pore network is homogeneous with respect to the pore diameters. The addition of spheres of different diameters then leads to the formation of hotspots within the pore network, depending on the concentration and diameter, as shown un Figure 7B. If only spheres are used as pore formers (Figure 7B,C), the diameter increases with a simultaneous reduction in the pore density compared to Figure 7A. If the spherical pore size is increased from 30 µm (Figure 7B) to 300 µm (Figure 7C), the density of the pore network is reduced and the hotspots form around the large spheres. The multimodal pore network combines the characteristics of the single pore networks and can be deliberately tuned by changing the ratio of the individual templates (Figure 7D).

The quantitative evaluation of the pore networks is shown in Figure 7E. In the investigated fiber sample with the maximum fiber content, the distance between the pores is <25 µm and increases to about 40 µm for the samples with the 30 µm spheres at a lower volume fraction. For the 300 µm spheres, the segment length increases to assume the maximum here. This correlates directly with the connectivity determined by the Euler number, see Figure 5C. If the number of connections of the branch nodes is considered, see Figure 7F, the fiber sample has the lowest number of connections, which directly correlates with the small segment length and thus distance. For the 30 + 300 µm spheres, there is a small number (<5) of nodes that have more than 20 connections to other pores, and the maximum values for connections <5 are lower than for the fiber sample. The fiber addition to the 30 + 300 µm spheres provides a small number of highly connected pores, however, consistent with the Euler number, this reduces the overall interconnectivity.

**Figure 8:**
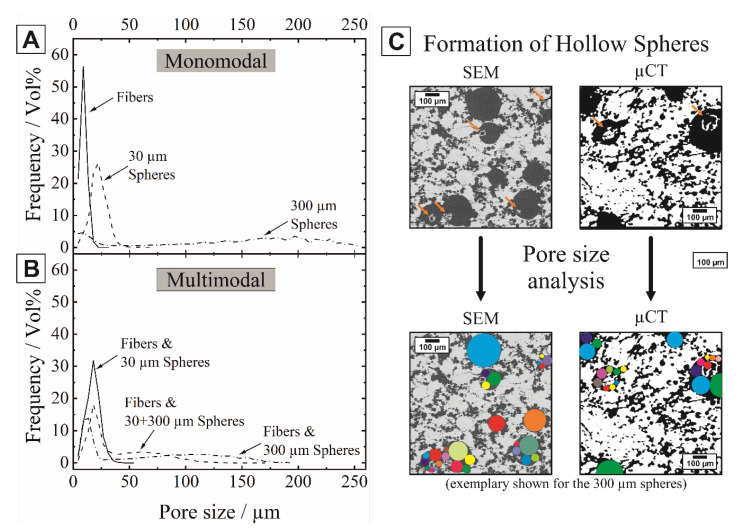
Pore size distributions of Al_2_O_3_ ceramics with monomodal (**A**) and multimodal (**B**) pore size distributions derived from µ-CT analysis. The formation of hollow spheres inside the spherical pores leads to a reduced pore size and broadening of the pore size distributions (**C**).

Figure 8 shows the µ-CT derived pore size distributions of the mono- and multimodal samples of the representative VOI. The corresponding Gaussian fitted mean pore sizes (d_50_ values) for one sample are summarized in Table 2. The µ-CT derived pore size distributions of the sintered Al_2_O_3_ ceramics partially overlap as a result of template-template interactions. Adjacent pore formers generate interconnected pore channels at high template loadings broadening the particle size distributions. The mean pore sizes of the monomodal fibrous samples (d_50_ = 9 µm) are in a good agreement with the initial fiber template size of 8 µm. The pore sizes of the monomodal samples with spherical pore formers showed slight deviations from the initial template size with 22 µm and 181 µm for the 30 µm and 300 µm spheres, respectively. This can be attributed to the formation of alumina hollow spheres within the spherical pores, which is significantly pronounced for the large 300 µm spherical templates shown in Figure 8C. Ceramic hollow spheres may form as an effect of the thermal debinding of the phenolic resin spheres. During the initial debinding stage, the phenolic resin spheres slightly expand and embed surrounding the ceramic material. At higher temperatures, they collapse and form a thin ceramic shell during complete burnout (T > 600 °C, see Figure 3), which afterwards shrinks and consolidates to an entrapped hollow sphere in the as-formed pore during the sintering stage. The formation of hollow spheres was also observed by other researchers using expandable microspheres [24]. The entrapped hollow spheres significantly decreased the pore size compared to the original template size. This decrease, however, could be accurately determined by the µCT-evaluation shown in the colored circles of Figure 8C, representing the real determined pore sizes. The multimodal alumina ceramics with bimodal mixtures of pyrolyzed cellulose fibers and 30 µm spheres exhibited a mean pore size of 18 µm due to the formation of interconnected pore channels between the organic templates. Mixtures containing fibers and 300 µm spheres showed bimodal pore size distributions due to the pronounced size difference of the sacrificial templates, characterized by two peaks at d^I^_50_ = 18 µm (fibrous pores) and a d^II^_50_ = 62 µm (spherical pores). For the trimodal mixtures containing all three types of pore formers, bimodal pore size distributions were also observed due to the overlap between the fibers and small spheres. The permeability calculation was performed on the digital twins of the samples.

Permeability requires a sufficient number of continuous connections from the inlet to the outlet side of the sample. This boundary condition is independent of the total connectivity and the connections per branch node. The monomodal addition of the pyrolyzed cellulose fibers did not lead to the formation of a fully permeable porous alumina matrix, even for a maximum possible amount of 34 Vol% C-fibers. The permeability and the pressure drop of porous ceramics are predominantly influenced by the total porosity, the pore size [48], and shape [5], the pore interconnectivity, the pore throats between the connected pores [15,49] and the tortuosity of the pore network. The distribution of 37 Vol% of 30 µm spheres in the Al_2_O_3_ matrix leads to a permeability of 4.1 D. For the larger 300 µm spheres a significant higher amount of 52 Vol% was required to obtain a permeable alumina matrix with a permeability of 9.0 D, resulting from the low degree of interconnectivity between the 10-times larger templates. In contrast, a significant increase of the permeability could be obtained for the bimodal samples combining the interconnected pore network of the pyrolyzed cellulose fibers with additional spherical pores for an improved flow-rate. Especially the combination of the 300 µm spheres with the fibers resulted in a permeability of 136.0 D, which is two magnitudes higher than the permeability of the samples with monomodal pore size distributions. The permeability of the multimodal porous alumina far exceeds the requirements for Diesel Particle Filters of 10^−11^–10^−12^ m^2^ (~1–10 D) [5]. The trimodal combination of templates (fibers, 30 and 300 µm spheres) could not further improve the permeability. The permeability of the multimodal porous alumina ceramics manufactured in this work were mainly dependent on the size of the pore formers as long as the porous matrix provided an interconnection between larger pores.

### 3.2. Mechanical Properties

**Figure 9:**
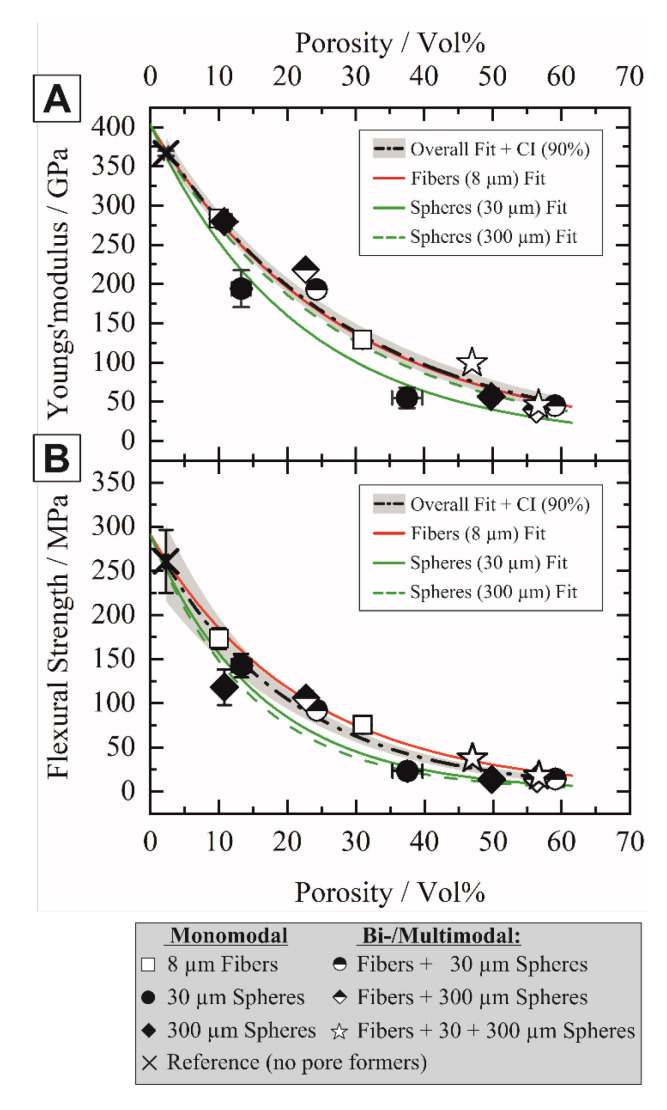
Influence of the amount and shape of the sacrificial templates on the mechanical properties of Al_2_O_3_ ceramics with multimodal porosity: Young’s modulus (**A**) and flexural strength (**B**) in dependence of the total sample porosity fitted by the exponential model of Equation (1).

The mono-, bi-, and trimodal distribution of anisotropic and isotropic pore formers significantly influenced the mechanical properties of the sintered alumina ceramics. Figure 9 shows the Young’s modulus (A) and flexural strength (B) in dependence of the total sample porosity for the mono- and multimodal porous ceramic samples with varying sphere to fiber ratios.

A general decrease in both flexural strength and Young’s modulus with increasing porosity was observed, which is directly dependent on the amount of each sacrificial template type. Numerous models have been developed to describe the strength-porosity dependence considering the influence of the pore size, shape, and interconnectivity [50]. The well-established exponential approaches from Duckworth [51] and Spriggs [52] were extended for non-spherical pores and can be described by Equation (1):

(1) $$M_{P}=M_{0}\cdot \exp\left(-bf_{p}\right),$$

where the mechanical property (here flexural strength and stiffness) of the porous material *M_P_* is given by the modulus of dense material *M*_0_, a dimensionless constant *b* ≈ 2–7 and the pore volume fraction *f_p_* [51,52]. The value of *b* is predominantly determined by the pore shape and orientation respective to the stress axis [53,54], which also could be confirmed by the results of this work. Both flexural strength and stiffness were mainly influenced by the type and size of the templates since the experimental data could not be well described by single overall curve fits (dash-dot black line with 90% confidence interval, Figure 9). We therefore used separate curve fits for the individual pore formers to highlight the tendencies. All samples containing fibrous pores (non- or half-filled symbols) exhibited higher stiffness and strength compared to the samples containing spherical pores (filled symbols), as shown in Figure 9. The small spherical pores caused significant lower stiffnesses (solid green line) in comparison to samples with fibrous pores (solid red line), while large spherical pores reduced the flexural strength the most (dashed green line). A reduced stiffness with a maintained strength is highly attractive to design strain-tolerant porous ceramics with a higher specific strength or higher thermal shock resistance [3,7]. A pore size dependent strength was frequently reported [12,55,56,57], and high strengths could be achieved with small pore sizes, matching with the results of this work. The origin of material failure is however not predominantly associated with the mean defect size, such as the mean pore size, but occurs at the most critical flaw. Especially under flexural loading, agglomerated pores (“clusters”) frequently caused the catastrophic brittle failure [15,58].

**Figure 10:**
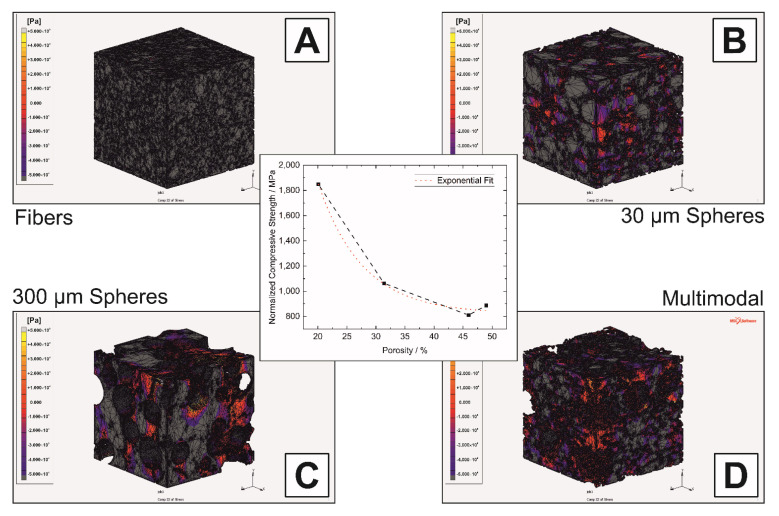
Stress distribution in σ_yy_ of the monomodal samples with fibrous (**A**), 30 µm (**B**) and 300 µm spherical pores (**C**) and the multimodal sample (**D**) with assigned displacement of 0.1% on top face; the corresponding compressive stress is shown in the middle. The legend shows the range from +500 MPa to −500 MPa.

This can also be observed by FEM calculations on the digital twin of the investigated structures, see plot of stress distribution σ_yy_ in loading direction y. When only fibers are used as pore formers, see Figure 10A, compressive stresses are predominant. As soon as the 30 + 300 µm spheres are used as pore-formers, tensile stresses occur at the pores, see Figure 10B. This was observed for both the 30 µm and the 300 µm pores, see Figure 10B–D. In particular, with the monomodally used spheres, an increase in tensile stresses can be observed here with increasing pore size from 30 to 300 µm in Figure 10C,D. Especially with the 300 µm spheres, which are larger by a factor of 10, tensile stresses occur predominantly here compared to the fiber-only structure.

In order to be able to exclude the influence of singular stress peaks, which are caused by strongly deformed, but unavoidable, FE elements, the observation of the accumulated compressive stresses and subsequently normalized to the corresponding number is carried out, see Figure 10 (middle). Depending on the porosity, an identical behavior of the exponential stress decrease can be observed compared to the experimental investigations. Based on the results from the FE simulations, which are in agreement with the experimental tests, the representative volume-of-interest can be set to a volume of 400 × 400 × 400 px^3^ or 896 × 896 × 896 µm^3^.

### 3.3. Thermal Properties

**Figure 11:**
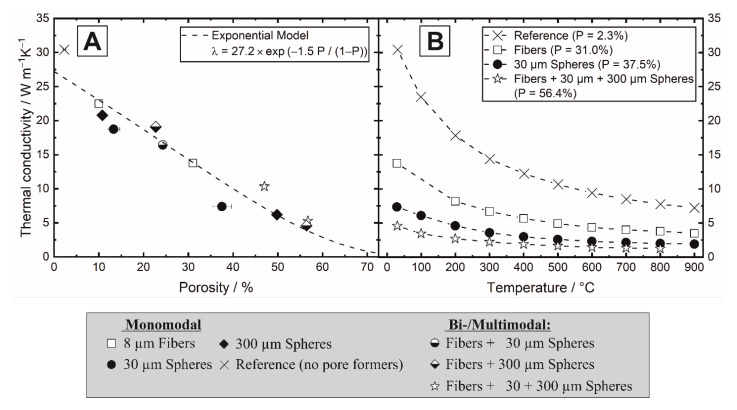
Influence of the total porosity (**A**) and pore former shape (**B**) on the thermal conductivity.

The thermal conductivity of the samples was determined using laser flash analysis between 25 and 900 °C. Figure 11A shows the thermal conductivity as a function of the porosity at 25 °C and Figure 11B shows the temperature dependent thermal conductivity for the selected samples. A general decrease of the thermal conductivity with both increasing porosity and increasing temperature was observed. The decrease in thermal conductivity with increasing temperature is caused by phonon-phonon scattering and the scattering of phonons on lattice defects and grain boundaries.

The porosity dependence of the thermal conductivity can be described with a modified exponential relation of Equation (2) [59]:

(2) $$\lambda_{P}=\lambda_{0}\cdot exp\left(\frac{-1.5\cdot f_{p}}{1-f_{p}}\right),$$

with the thermal conductivity of the porous (*λ_p_*) and dense (*λ*_0_) material and the total porosity *f_P_*. Equation (2) was applied to fit the experimental data of the thermal conductivity as a function of the total porosity. For the fitted λ_0_ = 27.2 Wm^−1^K^−1^, an acceptable fit of Equation (2) (R^2^ = 0.919) shows that corresponding to the mechanical properties, the thermal conductivity was not only dependent on the total porosity but was mainly influenced by the type, volume fractions and orientation of the sacrificial templates. Interestingly, the pore networks generated by monomodal 30 µm spheres (symbol: filled circles) induced significant lower thermal conductivities in the alumina matrix compared to the samples containing other templates or mixtures at similar porosities. This corresponds with the non- or low permeability of those samples. Bi- and trimodal samples containing high amounts of large 300 µm spheres showed increased thermal conductivities (symbols: half-filled diamond, left star), Figure 11. The thermal conductivity of all samples was measured parallel to the pressing direction, to which the tubular pores are perpendicular oriented. Based on the fiber alignment, an anisotropic thermal behavior can be expected with higher conductivities measured parallel to the fibrous pores [21,22].

## 4. Conclusions

Alumina ceramics with multimodal pore networks were successfully manufactured using fibrous and spherical sacrificial templates. α-Al_2_O_3_ powder mixtures containing different ratios of pyrolyzed cellulose fibers (anisotropic, aspect ratio l/d = 19) and phenolic resin spheres (isotropic, aspect ratio l/d = 1) were homogeneously mixed, uniaxially pressed, and sintered up to 1700 °C to extract the templates. The microstructure analysis revealed tubular and spherical pores in the alumina matrix with porosities of 0 to 60 Vol%. The influence of the pore-former volume fraction, shape and size was investigated and indicates general trends regarding the microstructure and mechanical/thermal properties of the multimodal porous Al_2_O_3_ ceramics:

- The application of the three Minkowski functionals (M1, M2, M3) to the µCT images of the heterogeneous structures with different sample formers in terms of shape and size was successfully applied. Thus, the representative volume-of-interest could be set to a size of 400 × 400 × 400 px^3^ or 896 × 896 × 896 µm^3^ at the available resolution of 2.24 µm and thus the digital twin could be defined. The digital twin of each structure enabled the visualization and evaluation of the pore network of the structures and the determination of their connectivity.
- The pyrolyzed cellulose fibers show a perpendicular alignment to the pressing direction induced by the uniaxial pressing. The combination of fibrous templates and spherical templates did not interfere with the alignment of the fibers in the samples with multimodal distributions of sacrificial templates. The volumetric evaluation using the digital twin for the orientation of the fibers has confirmed and complemented the 2D analysis.
- The permeability is mainly dependent on the pore size of the spherical pore formers as long as the porous matrix provides an interconnected pore network. The 300 µm phenolic resin spheres provided larger pore channels and thus a higher permeability in comparison to the 30 µm phenolic resin spheres. The tubular pores are essential to connect isolated spherical pores. The identified pore networks and their quantification by segment lengths and connectivity at the branch nodes are consistent with the results of the Euler number.
- The type of the sacrificial templates predominantly influenced the mechanical properties. Small tubular pores lead to a higher stiffness and strength compared to spherical pores, based on the smaller defect size and anisotropic microstructure. Low elastic moduli with higher specific strength were obtained for the samples with a monomodal distribution of 30 µm phenolic resin spheres. The FEM simulations performed on the digital twins agree with the experimental results with respect to the distribution of the stresses.

The multimodal combination of sacrificial templates and the resulting pore network with multimodal pore size distribution can be tailored regarding permeability, strength, and thermal conductivity for specific applications.

## Figures and Tables

**Table 1:** Compositions and corresponding target and total porosities of the realized alumina ceramics with monomodal, bimodal, and trimodal mixtures of sacrificial templates.

Batch No.	Distribution Type	Fibers /Vol%	30 µm Spheres /Vol%	300 µm Spheres /Vol%	Target Porosity /Vol%	Total Porosity /Vol%
1	Reference				0	2.3
2	Monomodal	10			10	10.0
3			15		15	13.3
4				15	15	10.8
5		34			34	31.0
6			37 *		37	37.5
7				52 *	52	49.8
8	Bimodal	8		16	25	22.2
9		8	16		25	24.3
10		22	35 *		57	59.1
11		17		50 *	67	56.4
12	Trimodal	28	8	8	44	47.0
13		5	27	27	58	56.7

* Maximum amount of spherical templates with sufficient green strength for handling and processing.

**Table 2:** µ-CT derived mean pore sizes (d_50_) and corresponding permeability of the sintered Al_2_O_3_ ceramics with representative mono-, bi- and trimodal pore distributions in comparison to the initial pore former size. The monomodal pore size distributions are characterized by a single peak, while bi- and trimodal samples containing 300 µm spheres exhibit bimodal pore size distributions (peak I and II).

Sample Type	Ratio of Sacrificial Templates/Vol%			Mean Pore Size ** (d_50_)/µm		Porosity * /Vol%	Permeability ** /D
	Fibers	35 µm Spheres	300 µm Spheres	Peak I (d^I^_50_)	Peak II (d^II^_50_)		(9.87·10^−13^ m^2^)
Initial pore formers *	100			8	-	-	-
		100		31	-	-	-
			100	303	-	-	-
Monomodal **	34			9	-	31.0	0
		37		22	-	37.5	4.1
			52	181	-	49.8	9.0
Bimodal **	22	35		18	-	59.1	34.9
	17		50	18	62	56.4	136.0
Trimodal **	5	27	27	18	53	47.0	33.4
	28	8	8	11	69	56.7	4.6

* Experimentally determined porosity and particle size distributions from SEM or laser diffraction. ** µ-CT derived pore sizes and permeability.

## Data Availability

Data sharing not applicable.
